# Vertical Transmission of Coxsackievirus A6 with Severe Congenital Pneumonia/Sepsis

**DOI:** 10.3390/ijerph20042843

**Published:** 2023-02-06

**Authors:** Ruka Nakasone, Miki Ogi, Aoi Kawamura, Osamu Miyake, Takumi Kido, Shinya Abe, Naoto Takahashi, Kandai Nozu, Kazumichi Fujioka

**Affiliations:** 1 Department of Pediatrics, Graduate School of Medicine, Kobe University, Kobe 650-0017, Japan; 2 Hyogo Prefectural Institute of Public Health Science, Kakogawa 675-0003, Japan; 3 Department of Pediatrics, Palmore Hospital, Kobe 650-0012, Japan; 4 Department of Pediatrics, The University of Tokyo Hospital, Tokyo 113-8655, Japan

**Keywords:** Coxsackievirus A6, vertical transmission, multiplex PCR, congenital pneumonia, HFMD

## Abstract

We report a case of vertical transmission of Coxsackievirus (CV)-A6 with severe congenital pneumonia/sepsis. A male infant presented with severe respiratory symptoms at birth and was treated with full cardiopulmonary support, including inhaled nitric oxide. Three days before delivery, his older brother was diagnosed with hand, foot, and mouth disease (HFMD). His mother developed transient fever 1 day before delivery and presented a blister on her thumb 2 days after delivery. A multiplex polymerase chain reaction test on day 2 was positive for human rhinovirus/enterovirus. CV-A6 was later detected in the serum, tracheal aspirate, and stool of the patient sampled on day 6, and in the maternal serum sampled on the day of delivery. He was diagnosed with congenital CV-A6 pneumonia/sepsis caused by vertical transmission, based on VP1 consensus sequences used for typing of the virus that demonstrated a 100% match between the mother and infant. Further, the strain was closely related to the lethal CV-A6-Changchun strains in the phylogenetic analysis of the P2 region, which contributes to the pathogenicity. In conclusion, congenital CV-A6 infection should be considered if a woman exhibits HFMD symptoms during the perinatal period. Detailed virologic examination is useful for understanding its pathogenesis.

## 1. Introduction

Coxsackievirus (CV)-A6, a human enterovirus (EV) [1], has been identified as the primary causative virus of hand, foot, and mouth disease (HFMD) in Japan since 2011 [2]. Congenital EV infection is severe [1]; however, there are few reports on the vertical transmission of CV-A6 [3,4]. Here, we report a case of congenital CV-A6 pneumonia/sepsis caused by maternal infection after HFMD in an older sibling.

In addition, through detailed virological studies, we demonstrate that the causative virus has a genetic background similar to that of the CV-A6 strains isolated from patients with HFMD in northeast China in 2013, which were highly virulent [5].

## 2. Detailed Case Description

A 34-year-old woman, gravida 2, para 1, who had been treated for gestational diabetes with insulin therapy, delivered a male infant (birth weight, 3176 g; appearance, pulse, grimace, activity, and respiration scores, 8 at 1 min and 8 at 5 min) at 40 and 5/7 weeks of gestation. Three days before delivery, her older son was diagnosed with HFMD, with a fever and typical rash. She developed a transient fever 1 day before delivery and presented a 2 mm diameter blister on her thumb 2 days after delivery. At delivery, her laboratory data demonstrated an increased C-reactive protein (CRP, 6.60 mg/dL) level, normal white blood cell (WBC, 6900/µL) count, and negative polymerase chain reaction (PCR) for severe acute respiratory syndrome coronavirus 2 (SARS-CoV-2). The infant cried and breathed spontaneously immediately after birth, and early skin-to-skin contact was initiated. He began grunting and his oxygen saturation (SpO_2_, measured by pulse oximetry) levels persisted under 60% on pulse oximeter; thus, oxygen supplementation was initiated 3 min after birth. Continuous positive airway pressure with 100% oxygen was initiated because his SpO_2_ levels did not increase with oxygen administration alone; however, the levels persisted below 95%.

**Figure 1:**
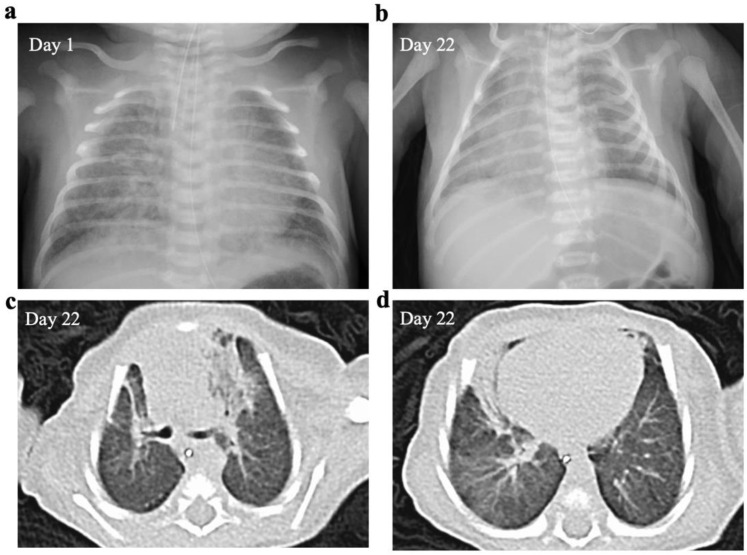
Chest radiograph and computed tomography (CT) scan. The chest radiograph displays bilateral hazy opacities (**a**). The chest radiograph (**b**) and CT examination (**c**,**d**) performed at 22 days of age reveal infiltrative shadows in both lung fields and decreased lung capacity.

The infant was transferred to our hospital 3 h after delivery because of severe respiratory failure. He was intubated immediately after admission for hypoxia and respiratory distress accompanied by grunting, retractions, and tachypnea. On admission, no abnormal physical signs suggestive of infection were noted, including fever or rash, except tachycardia. His chest radiograph indicated bilateral hazy opacities (Figure 1a); however, laboratory data demonstrated only a minor increase in the WBC count (16,200/µL) and no increases in CRP (0.05 mg/dL) or immunoglobulin M (7 mg/dL) levels.

**Figure 2:**
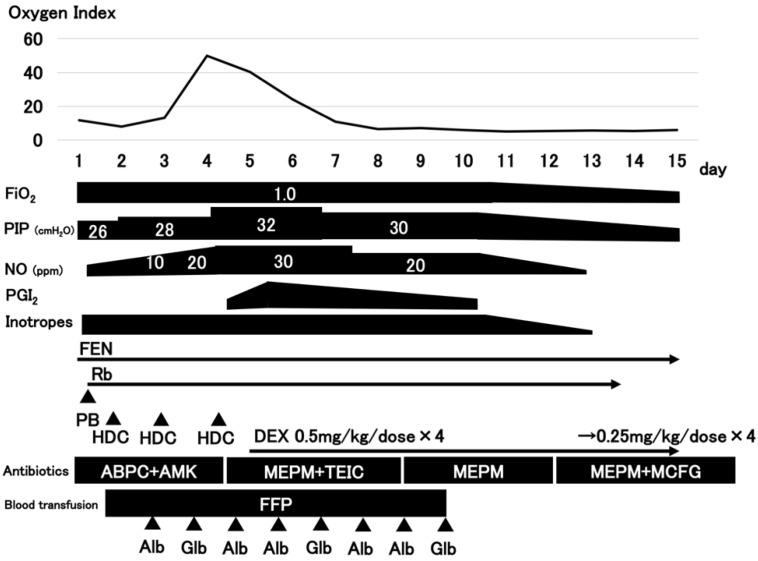
The clinical course of the patient. FiO_2_, fraction of inspired oxygen; PIP, peak inspiratory pressure; NO, nitric oxide; PGI_2_, prostaglandin I_2_ (epoprostenol); Inotropes (DOA, dopamine; DOB, dobutamine); FEN, fentanyl; Rb, rocuronium bromide; PB, phenobarbital; HDC, hydrocortisone; DEX, dexamethasone; ABPC, ampicillin; AMK, amikacin; MEPM, meropenem; TEIC, teicoplanin; MCFG, micafungin; FFP, fresh frozen plasma; Alb, albumin; Glb, globulin.

The clinical course of the patient is depicted in Figure 2. The patient was diagnosed with persistent pulmonary hypertension of the newborn (PPHN) using echocardiography 7 h after admission. We initiated inhaled nitric oxide (iNO) therapy along with deep sedation with fentanyl and muscle relaxants, inotropes, hydrocortisone, and fresh frozen plasma. Further, we administered antibiotics (ampicillin and amikacin) because we could not exclude the possibility of pneumonia. Epoprostenol sodium and dexamethasone were initiated on day 4 because of worsening respiratory distress, in addition to a 10 ppm to 30 ppm increase in iNO. Oxygenation gradually improved after day 5, iNO therapy was terminated on day 13, and the patient was extubated on day 15.

Chest radiograph and CT examination performed at 22 days of age revealed infiltrative shadows in both lung fields and decreased lung capacity (Figure 1b–d). He required noninvasive positive pressure support until day 37 and was discharged on day 49 on home oxygen therapy, which was terminated at 4 months of age. His psychomotor development was normal and corresponded to his age (sitting at 7 months).

A multiplex PCR test on day 2, which was routinely performed for the differential diagnoses of SARS-CoV-2 in patients with severe respiratory symptoms, was positive for human rhinovirus/enterovirus (Table 1).

Bacterial cultures collected on admission were negative in all specimens. CV-A6 was later detected in the patient’s serum, tracheal aspirate, and stool collected on day 6 (negative for cerebrospinal fluid on day 27) and in the maternal serum collected on the day of delivery by CODEHOP VP1 reverse transcription-seminested PCR [6] and sequence analysis (Table 2).

**Figure 3:**
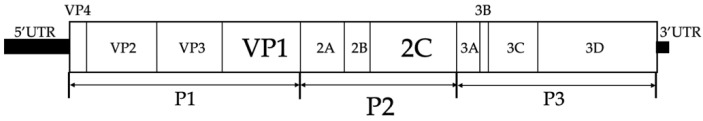
Structure of the enterovirus genome. The genome contains a single open reading frame that encodes a polyprotein and is flanked by 5′ and 3′ untranslated regions (UTRs). The encoded polyprotein contains three major regions (P1, P2, and P3). These regions can self-digest into four separate structural proteins (VP1, VP2, VP3, and VP4 from P1) and seven nonstructural proteins (2A, 2B, and 2C from P2; 3A, 3B, 3C, and 3D from P3) [5].

**Figure 4:**
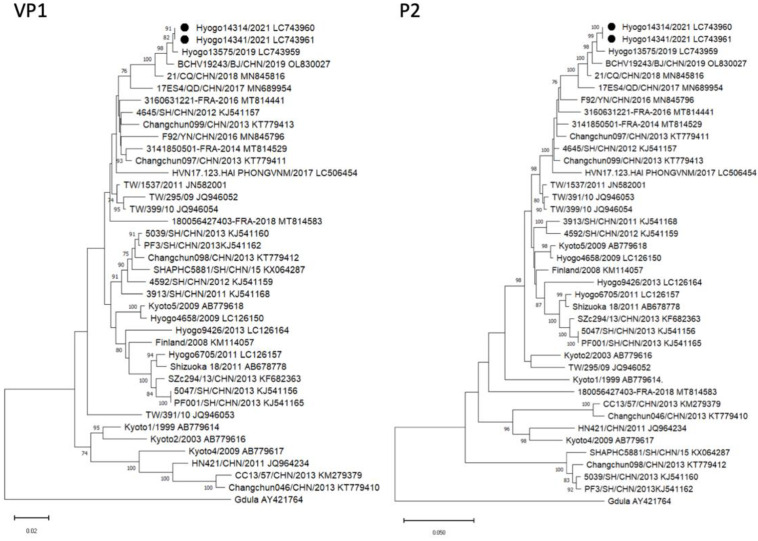
Phylogenetic analysis of the VP1 and P2 regions of the CV-A6 strains. (**Left**) Neighbor-joining tree based on the VP1 sequences (positions 2441 to 3355 correspond to the Gdula genome). (**Right**) Neighbor-joining tree based on the P2 sequences (positions 3356 to 5089 correspond to the Gdula genome). Both phylogenetic trees were tested using the bootstrap method for 1000 replicates, and values of >70 are shown at the nodes. ●, CV-A6 Hyogo strains of this case.

He was diagnosed with congenital CV-A6 pneumonia/sepsis caused by vertical transmission, based on the full length of the VP1 region that demonstrated a 100% match between the mother (Hyogo14341: GenBank accession no. LC743961) and infant (Hyogo14314: GenBank accession no. LC743960). Phylogenetic analysis of the VP1 region indicated that these strains were closely related to the strains detected in Hyogo Prefecture and China from 2017 to 2019 (Figure 3 and Figure 4). In addition, a detailed analysis of the P2 region, which is most strongly correlated with the lethality of CV-A6, identified Hyogo14341 and Hyogo14134 strains as belonging to a cluster similar to the lethal CV-A6 Changchun strains (Changchun046, Changchun097, and Changchun099) and distant from the nonlethal strain (Changchun098) (Figure 3 and Figure 4).

Furthermore, all eight amino acid substitutions that differed between the lethal and nonlethal groups were identical to those of Changchun097 and Changchun099, including three substitutions (324, 339, and 556) in the 2C region that matched the three lethal strains (Changchun046, Changchun097, and Changchun099) (Table 3).

The serum cytokine profiles on day 1 were consistent with those of a viral infection (increased interleukin-6 (IL-6), interleukin-8 (IL-8), granulocyte-colony stimulating factor (G-CSF), interferon-γ (IFN-γ), and tumor necrosis factor-α (TNF-α) levels; Table 4).

## 3. Discussion

CV-A6 is a recognized causative agent of herpangina; however, it has been reported as a new causative agent of HFMD in Asia and Europe since 2008 [1]. In Japan, the detection rate of CV-A6 in HFMD cases has increased since 2009. CV-A6 has become the leading cause of HFMD, surpassing the epidemics of EV 71 and CV-A16, which were the primary causes [2]. However, there have been few reports of neonatal CV-A6 infection. Bruning et al. reported on an extremely preterm twin case of neonatal CV-A6 infection, which was horizontally transmitted and presented mild phenotypes [3]. Similarly, Xu et al. reported on a case series of 16 neonatal CV-A6 infections, all of which demonstrated horizontal transmission and mild phenotypes [4]. A systematic review of severe neonatal EV infections identified CV-B, echovirus, and EV 71 as the causative viruses; however, it did not include CV-A6 [7]. The risk factors for severe neonatal EV infection were prematurity, perinatal maternal infection, onset within the first week of life, and the lack of specific antibodies [1]. Our case had two of these risk factors.

We considered two factors as the causes of severity. First, the viral infection (the onset of HFMD) occurred immediately before delivery; therefore, insufficient maternal neutralizing antibodies were produced against CV-A6, leading to vertical infection in the absence of transferred antibodies in the fetus and the development of fulminant symptoms. Based on the findings of a SARS-CoV-2 study, the production of neutralizing antibodies requires at least 2 weeks; thus, in our case, the fetus most likely did not have adequate immunity against CV-A6 [8]. Second, a report from China detailing the virologic examination of CV-A6-Changchun viruses, which are highly pathogenic and lethal strains, revealed that the 2C protein region is central to the pathogenicity of CV-A6 strains [5]. Notably, after a phylogenetic study, we confirmed that our strains (Hyogo14314 and Hyogo14341) were highly concordant with the Changchun strains. Thus, the virulence of the strains alone may have also contributed to the aggravation of the disease.

Moreover, in this case, the serum cytokine profile was measured in residual sera at 1, 4, and 10 days of age, and the IL-6, IL-8, G-CSF, IFN-γ, and TNF-α levels significantly increased at 1 day of age. In a review, Zhang et al. reported on elevated IL-6, IL-8, G-CSF, IFN-γ, and TNF-α in severe EV 71 infection; thus, our results were consistent with the findings of severe EV infection [9].

This case highlighted two aspects. First, CV-A6 can cause vertical infection, severe congenital pneumonia, and PPHN. Second, multiplex PCR can assist in the diagnosis (maternal information and surrounding infections) of congenital pneumonia of an unknown cause.

## 4. Conclusions

In conclusion, congenital CV-A6 infection should be considered if a woman exhibits symptoms of HFMD during the perinatal period. Detailed virologic examination is useful for understanding the pathogenesis.

## Figures and Tables

**Table 1:** Multiplex polymerase chain reaction results.

Virus/Bacteria Type	Result
Adeno virus	Negative
Human coronavirus (HCoV)-229E	Negative
HCoV-HKU1	Negative
HCoV-NL63	Negative
HCoV-OC43	Negative
Severe acute respiratory syndrome coronavirus 2	Negative
Human metapneumovirus	Negative
Human rhinovirus/enterovirus	Positive
Influenza virus A	Negative
Influenza virus B	Negative
Parainfluenza virus 1	Negative
Parainfluenza virus 2	Negative
Parainfluenza virus 3	Negative
Parainfluenza virus 4	Negative
Respiratory syncytial virus	Negative
Bordetella pertussis	Negative
Chlamydophila pneumoniae	Negative
Mycoplasma pneumoniae	Negative

**Table 2:** Bacterial and viral examination results.

	Specimen	Day	Result
Bacterial culture	Neonatal serum	Day 1	Negative
	Tracheal aspirate	Day 1	Negative
	External ear	Day 1	Negative
	Pharyngeal	Day 1	Negative
	Umbilical cord	Day 1	Negative
	Cerebrospinal fluid	Day 27	Negative
PCR	Maternal serum	Day 0	CV-A6
	Serum	Day 6	CV-A6
	Tracheal aspirate	Day 6	CV-A6
	Pharyngeal	Day 6	Negative
	Urine	Day 6	Negative
	Stool	Day 6	CV-A6
	Cerebrospinal fluid	Day 27	Negative

CV-A6, Coxsackievirus-A6; PCR, polymerase chain reaction.

**Table 3:** Alignment of amino acid sequences of the P2 region of CV-A6 Hyogo strains with the lethal and nonlethal strains.

Virus Type	129	262	267	290	297	324	339	556
Changchun098/CHN/2013_nonlethal	G	S	L	R	N	V	F	I
Changchun046/CHN/2013_lethal	N	S	L	K	S	A	Y	V
Changchun097/CHN/2013_lethal	N	N	F	R	N	A	Y	V
Changchun099/CHN/2013_lethal	N	N	F	R	N	A	Y	V
Hyogo14314 (LC743960)	N	N	F	R	N	A	Y	V
Hyogo14341(LC743961)	N	N	F	R	N	A	Y	V

**Table 4:** Results of the serum cytokine profiles.

	Day 1	Day 4	Day 10
IL-1b	3.1	0.3	OOR<
IL-2	OOR<	OOR<	OOR<
IL-4	1.28	OOR<	OOR<
IL-5	OOR<	OOR<	OOR<
IL-6	1190	178	OOR<
IL-7	OOR<	OOR<	OOR<
IL-8	193	119	75
IL-10	9.85	3.37	OOR<
IL-12	OOR<	OOR<	OOR<
IL-13	OOR<	OOR<	OOR<
IL-17	2	0.18	OOR<
G-CSF	2208	299	OOR<
GM-CSF	OOR<	OOR<	OOR<
IFN-γ	50.6	27.3	OOR<
MCP-1	693	577	15
MIP-1β	129	151	83
TNF-α	29.1	31.7	8.1

IL, interleukin; G-CSF, granulocyte-colony stimulating factor; GM-CSF, granulocyte–macrophage-colony stimulating factor; IFN-γ, interferon-γ; MCP-1, monocyte chemotactic protein-1; MIP-1β, macrophage inflammatory protein-1β; TNF-α, tumor necrosis factor-α; and OOR<, out of range or below.

## Data Availability

Not applicable.
